# Extracorporeal organ support and the kidney

**DOI:** 10.3389/fneph.2022.924363

**Published:** 2022-08-05

**Authors:** Maria-Jimena Muciño-Bermejo

**Affiliations:** ^1^ Intensive Care Unit, The American British Cowdray Medical Center, Mexico City, Mexico; ^2^ International Renal Research Institute of Vicenza (IRRIV), Vicenza, Italy; ^3^ Health Sciences Department, Anahuac University, Mexico City, Mexico; ^4^ Medical Division, Medecins SansFontières – OCBA (Operational Centre Barcelona-Athens), Barcelona, Spain

**Keywords:** extracorporeal organ support, acute kidney injury, hemodialysis, hemofiltration, extracorporeal membrane oxygenation, albumin dialysis

## Abstract

The concept of extracorporeal organ support (ECOS) encompasses kidney, respiratory, cardiac and hepatic support. In an era of increasing incidence and survival of patients with single or multiple organ failure, knowledge on both multiorgan crosstalk and the physiopathological consequences of extracorporeal organ support have become increasingly important. Immerse within the cross-talk of multiple organ failure (MOF), Acute kidney injury (AKI) may be a part of the clinical presentation in patients undergoing ECOS, either as a concurrent clinical issue since the very start of ECOS or as a *de novo* event at any point in the clinical course. At any point during the clinical course of a patient with single or multiple organ failure undergoing ECOS, renal function may improve or deteriorate, as a result of the interaction of multiple factors, including multiorgan crosstalk and physiological consequences of ECOS. Common physiopathological ways in which ECOS may influence renal function includes: 1) multiorgan crosstalk (preexisting or *de-novo* 2)Hemodynamic changes and 3) ECOS-associated coagulation abnormalities and 3) Also, cytokine profile switch, neurohumoral changes and toxins clearance may contribute to the expected physiological changes related to ECOS. The main objective of this review is to summarize the described mechanisms influencing the renal function during the course of ECOS, including renal replacement therapy, extracorporeal membrane oxygenation/carbon dioxide removal and albumin dialysis.

## Graphical Abstract

Extracorporeal organ support (ECOS) was developed in order to support a failing organ and to prevent and/or reverse secondary multiorgan failure. ECOS provide extensive physiological benefits. However, both multiorganic crosstalk and patient interaction with the extracorporeal support may have renal repurcussions. Kidney vulnerability is the result of both kidney frailty (i.e. preexisting kidney/organic damage) and the summatory of adverse renal events during the clinical course (i.e hipoxia+nephrotoxicity+Hypervolemia). The present manuscript emphasizes on both the shared physiopathological pathways and individual differences to be taken into account for different to be taken into account for different kind of ECOS (renal replacement theraphy, extracorporeal membrane oxygenation, and albumim dialysis) as well as kidney vulnerabilty points to be taken into account in order to reach favorable clinical outcomes during ECOS. No, nitro oxide; ROS, reactive oxygen species; ECMO, Extracorporeal Membrane Oxigenation; RRT, Renal Replacement Theraphy; BNP, Brain Natriuretic peptide.

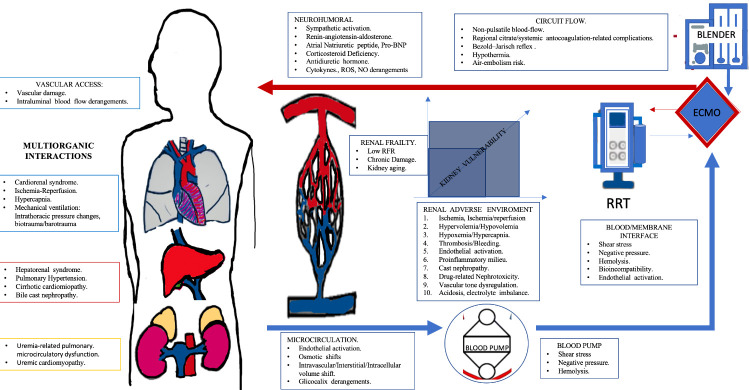

## Introduction

Extracorporeal organ support (ECOS) was developed in order to support a failing organ maintaining homeostasis as a bridge to recovery or transplantation (1).

However, both multiorgan crosstalk and the physiological consequences of ECOS (including hemodynamic changes, coagulation derangements and cytokine and neurohormonal profiles switch) may lead to adverse renal repercussions, In the following paragraphs, a review on the mechanisms that may give place to adverse renal repercussions during ECOS will be made (**Graphical Abstract**).

## Renal replacement therapy

### Renal replacement therapy and multiorgan crosstalk

In patients with precedent chronic kidney disease before initiation of CRRT or a second form of ECOS, preexisting physiological derangements and organic crosstalk must be contemplated. Chronic kidney disease, (CKD) and AKI are closely interconnected; CKD increases the kidney vulnerability to AKI, and AKI may worsen preexisting CKD, increasing the odds of end-stage kidney disease (2) (3).At a cellular level. Endoplasmic reticulum (ER) is a major component in the control of protein synthesis, fold, and degradation *via* the unfolded protein response pathway. Many pathologies (i.e. hypoxia), induce a maladaptive unfolded protein response pathway called ER stress on kidneys, giving place to both AKI and CKD. Tubular inflammatory pathways may be triggered specifically by the interaction of mitochondrial DNA with the endoplasmic reticulum membrane, initiating the continuance of tubular inflammation in AKI. Also, mitochondrial injury gives place to abnormal tubular inflammation, a major fibrotic contributor (4, 5).

At a tissular and organ level, CKD contributes to vascular remodeling that implies endothelial dysfunction, decreased bioavailability of nitric oxide, fluid overload, and shunting *via* arterio-venous fistulae, developing pulmonary hypertension (6). Additionally, uremia-related pulmonary microcirculatory dysfunction, may give place to a restrictive, poorly compliant lung (7).

Accelerated coronary artery atherosclerosis is linked toto CKD, through elevated blood pressure, dyslipidemia, altered calcium/phosphorus metabolism, vascular remodeling and augmented vascular stiffness (2, 8). Uremic cardiomyopathy is linked to biventricular hypertrophy, systolic and diastolic dysfunction, capillary rarefication and heart fibrosis (9, 10).

### Renal replacement therapy-related hemodynamic changes

Improvement of hemodynamic status during acute renal replacement therapy (aRRT) is linked to: 1)Correction of volume overload and pulmonary edema, 2) Correction of electrolyte and acid-base disorders and 3) Removal of cardio depressant molecules, including tumor necrosis factor-α, proinflamatory interleukins, pathogen-associated molecular patterns and damage-associated molecular patterns (11–13).

Hemodynamic instability during renal replacement therapy (HIRRT) occurs in 19–43% of CRRT treatments, may be associated to any RRT modality, impair renal recovery and is linked to increased mortality (12–16)

HIRRT may be the result of any possible interactions between cardiac output alterations, impaired systemic vascular resistances and endothelial derangements (12, 13):

− Diminished cardiac preload caused by of ineffective mobilization of unstressed blood volume (due to ineffective venoconstriction and/or fluid leak into the interstitium) and can be further increased by Bezold–Jarisch reflex, resulting in loss of peripheral sympathetic vasoconstriction (12, 13).

Other factors related to altered cardiac output include reduced preload due to absolute hypovolemia, increased preload and cardiac dysfunction because of absolute volume overload, and dysregulation of cardiac contraction and electric stability because of acidosis and/or electrolyte disturbances (12, 13):

− During regional citrate anticoagulation, infused citrate is metabolized in the liver and muscle to be fed into the Krebs cycle, producing 3 mmol of bicarbonate for each mmol of trisodium citrate produced; Dyalisate fluid with low concentration of bicarbonate is used. If a very small amount of citrate load is infused, it may give place to a negative bicarbonate balance, and thus metabolic acidosis. Ineffective citrate metabolism does not produce bicarbonate, leading to a negative buffer balance (12, 13).

− When regional citrate anticoagulation is used, calcium is removed as the citrate-calcium complex is replaced to keep homeostasis, inadequate supplementation is related to hypocalcemia (12, 13). Ionized calcium bellow <1.0 mmol/L independently predicts mortality in critically ill patients on aRRT; lower dialysate calcium concentration is associated with sudden cardiac arrest (17);

Hypothermia, a myocardial depressor may develop, being continuous veno-venous modalities, dialysate temperature, dialysate flow and blood flow contributors. Mild hypothermia prevent hypotension by inducing vasoconstriction but is also associated with myocardial stunning. Body temperature <30°C augments cytoplasmatic calcium and cyclic adenosine monophosphate, diminishing calcium sensitivity of contractile proteins, impairing the sensitivity of adrenergic receptors to agonists (18, 19).

Myocardial stunning during RRT, is related to reduced regional myocardial perfusion, (even without coronary artery disease), independently from hypovolemia inflammatory mediators released during bioincompatibility responses and activated complement components modify leukocyte responses and deranges blood vessel homeostasis (18, 19).

Glycocalyx is determinant in the homeostasis in the capillary barrier, interacting to regulate vascular tone; Sepsis and volume overload alters the glycocalyx and increase vascular permeability. Glycocalyx impairment alters capillary refilling and osmotic shift, important components in the development of hemodynamic derangements during aRRT Moreover, AKI and RRT *per se* damage the capillary barrier and microcirculation (18–20).

### Renal replacement therapy-related homeostatic derangements

Even without RRT, biomarkers of clot formation may be increased during AKI, as procoagulant stimulus may be a part of sepsis and the inflammatory response caused by the release of monocyte tissue factor of any cause (21, 22).

Aggregometric analyses described among patients undergoing CRRT include diminished arachidonic acid-induced platelet aggregation after 6 hours within the beginning of CRRT. and progredient increase of clot firmness (23, 24).

Blood-circuit interactions itself play a significant role on thrombogenesis during CRRT (24):

Damage to the distal catheter tip and surrounding tissues activates the coagulation pathway. Intrathoracic catheters may diminish its blood flow if supra cardiac central filling pressures are low or if the patient is hypovolemic and is sat in the upright position. 1.

Intrinsic coagulation pathway activation may be triggered by the rapid absorption of plasma proteins onto the hemofilter surface.2.

Platelet activation result from turbulent blood flow, contact with air and non-biological materials, blood cells damage in the roller pump.3.

Biocompatibility of venous catheter, incorrect assembly of circuit components and residual pockets of air during circuit priming.4. Filtration fraction greater than 30% may increase the probability of haemoconcentration and clotting inside haemofilter; predilution permits larger ultrafiltrate volumes without increasing the filtration fraction.

Also, both AKI and RRT have been reported to be associated with significant bleeding:

1. On selected ICU populations, bleeding is reported to occur in 48% of AKI patients but in 57% of non-AKI patients (25).

2. Regional citrate anticoagulation is associated with fewer bleeding complications, and longer filter life span (26).

3. Anticoagulant-related nephropathy may be seen in patients receiving warfarin, direct thrombin or a factor Xa inhibitors. Over anticoagulation causes profuse glomerular haemorrhage; histopathology shows numerous renal tubules filled with red cells and red cell casts (27–29).

## Extracorporeal membrane oxygenation

### Multiorgan crosstalk considerations in ECMO

Hypoxia, ischemia-reperfusion, cardiorenal interactions, lung-kidney interactions and mechanical ventilation consequences, altogether, must be taken into account to overcome kidney vulnerability points during ECMO (10).

Kidney hypoxia gives place to tubular ER stress and the maladaptive unfolded protein response pathway, contributing to phenotypic changes of renal tubular damage (30). Also, ischemia/reperfusion, oxidative stress and diminished NO bioavailability influences medullary perfusion, as vasa recta are highly reactive to vasoactive substances, including angiotensin II, adenosine, endothelin-1, reactive oxygen species (ROS), and NO.

After ischemia/reperfusion, differential excretion of vasoactive substances may create an imbalance between vasodilation and vasoconstriction; after hypoxia/reoxygenation, descending vasa recta has an increased reactivity to angiotensin II (31). As renal medullary blood flow depends on vasa recta and its particular oxygen and metabolic condition, renal medulla is extremely vulnerable to hypoxia. After severe ischemia the renal medullary blood flow may display delayed recovery (31).

Before ECMO is started, diminished cardiac output, elevated intra-thoracic pressure, hypercapnia, systemic inflammation/immune-mediated effects, and neurohormonal derangements may contribute to kidney damage. If heart failure is present, fluid retention, positive fluid balance, intra-abdominal pressure increase and renal congestion contribute to renal blood flow derangements and cardiorenal syndrome. Also, critical care related complications, (limb ischemia, infection, and coagulopathy) contribute to AKI (32, 33).

Mechanical ventilation itself give place to hemodynamics derangement and release of pro-inflammatory cytokines, being plasmatic cytokine levels predictive of AKI and non-recovery of kidney function (34). Additionally, activation of the sympathetic and renin–angiotensin–aldosterone systems and suppression of atrial natriuretic peptide may induce positive fluid balance (32–34).

Positive end-expiratory pressure may increase intrathoracic pressure, reducing venous return and cardiac output, augmenting right ventricular afterload, systemic venous pressure, venous congestion, and ultimately diminishing renal perfusion. Also, permissive hypoxia and hypercapnia might ensue and decrease renal blood flow (32–35)

Not surprisingly, risk factors for AKI during ECMO include post cardiotomy shock, late ECMO initiation, reduced left ventricle ejection fraction, intraoperative transfusion, high lactate, elevated neutrophil to lymphocyte ratio, and elevated inotropic equivalents (32–34) and **Table 1**.

**Table 1:** Extracorporeal Organ Support (ECOS) systems and its kidney damage physiopathological mechanisms.

ECOS system	Multiorgan crosstalk.	Hemodynamic changes.	Coagulation abnormalities	Cytokine profile changes.
RRT	-In patients with preexisting CKD, consider the possibility of preexisting endothelial dysfunction, pulmonary hypertension, coronary artery atherosclerosis, biventricular hypertrophy, systolic and diastolic dysfunction, capillary rarefication and heart fibrosis (7, 9, 10).	-Hemodynamic instability during RRT: -Diminished cardiac preload caused by of ineffective mobilization of unstressed blood volume and Bezold–Jarisch reflex (12, 13)., -In citrate regional anticoagulation, beware of bicarbonate and hypocalcemia (12, 13, 17). -Hypothermia leads to cardiac depression (18, 19) -Myocardial stunning during RRT (18, 19) -Glycocalyx derangements (18–20)	-Diminished arachidonic acid-induced platelet aggregation and progredient increase of clot firmness (23, 24). -Intrinsic coagulation pathway activation (24).. -Platelet activationfrom turbulent blood flow and contact with air and non-biological surface (24).. -Anticoagulant-related nephropathy (27–29).	-Use of adsorption membranes, sorbent cartridges or columns during ensue removal of proinflammatory cytokines and endotoxins early in septic shock. improve clinical outcomes in septic shock, (MAP, norepinephrine inotropic score and vasopressor dependency index (36) (37) (38)..
ECMO	-Kidney hypoxia gives place to tubular ER stress and the maladaptive unfolded protein response pathway (30) - Diminished cardiac output, elevated intra-thorcic pressure, hypercapnia, systemic inflammation/immune-mediated effects (32, 33) -Activation of the sympathetic and renin–angiotensin–aldosterone systems and suppression of atrial natriuretic peptide (33, 34). -Positive end-expiratory pressure may increase intrathoracic pressure, reducing venous return and cardiac output, augmenting right ventricular afterload, systemic venous pressure, venous congestion, and ultimately diminishing renal perfusion (32–35)	-GFR is diminished and renal vascular resistance at the preglomerular afferent site is augmented in heart failure with conserved and reduced ejection fraction (39). -Elevated central venous pressure increase intratubular pressure, counteracting GFR (40) -Nonpulsatile perfusion diminishes hemodynamic energy resulting in capillary collapse, microvascular shunting, and release of inflammatory mediators (41) (42).	-Blood contact with nonendothelial surfaces that leads to coagulation and fibrinolytic activation and a complement-mediated inflammatory response (43) (44). -Thrombus Deposition (Circuit /Oxygenator). (44) -Pump Thrombosis) (44). -Heparin-induced thrombocytopenia. ) (44) -Hemolysis) (44) -Thrombocytopenia (44). -Hyperfibrinolysis (45). -Disseminated Intravascular Coagulation (44). -Acquired von Willebrand syndrome (44).	-Similar to the cytokine-mediated inflammatory response to cardiopulmonary bypass (mainly interleukins 1,6,8 and tumoral necrosis factors), inflammatory response to ECMO consists activation of the complement system and endothelial activation (46–48); -Hemoadsorption during ECMO with good clinical results have been reported (48)
Extracorporeal liver support.	-Hepatorenal syndrome (49,50) -Renal manifestations of viral hepatitis (50–52). -Cirrhotic cardiomyopathy, cardiorenal syndrome (50–52), -Corticosteroid deficiency (50–52),	Portal hypertension: Increased intrahepatic resistance, splanchnic vasodilatation, and portosystemic collateral vessels (53) MARS diminishes hyperdynamic circulation (decrease in CO, increased MAP and SVRI), with no significant changes in pulmonary arterial pressure and capillary pulmonary pressures. (54).	-Bleeding and thrombotic complications are described in g both patients undergoing MARS, and patients with liver failure (55–57) -After MARS PT-INR, aPTT, FDP, and D-dimer significantly increased, while platelet and fibrinogen diminished. MARS also reduced coagulation factors, especially factor V (56).	Liver failure implies accumulation of a broad spectrum of water-soluble and lipophilic toxins and immune mediators (58, 59). -Oxidized forms of albumin and cytokines, are key factors in the pathogenesis of AOCLF (58, 59). -MARS (DIALIVE) have been described to diminish total bilirubin, biliary acids, BUN, ammonia, TNF-alpha, IL-6, and IL-1beta (60, 61)

RRT, Replacement Renal therapy; ECMO, Extracorporeal Membrane oxygenation; CKD, Chronic kidney disease; GFR, glomerular filtration rate; AOCLF, Acute on chronic liver failure.

### ECMO-related hemodynamic changes

Hypoxemia-related pulmonary hypertension pulmonary edema, systemic inflammation and cardiac failure seems to be the main contributing factor to ECMO-associated kidney disfunction, while ECMO-induced hemodynamic changes contribute to perpetuate kidney damage (19, 62, 63):

1. Renal dysfunction is related to left ventricular geometry alterations, diminished midwall fractional shortening, and elevated NT‐proBNP (64).

2. GFR is diminished and renal vascular resistance at the preglomerular afferent site is augmented in heart failure with conserved and reduced ejection fraction (39).

3. Elevated central venous pressure increase intratubular pressure, counteracting glomerular filtration rate (GFR) (40)

ECMO itself may contribute to hemodynamic derangements affecting kidney function (65):

1. Pulsatile blood flow is important to maintain renal cortical blood flow; Nonpulsatile perfusion diminishes hemodynamic energy resulting in capillary collapse, microvascular shunting, and release of inflammatory mediators (41, 42).

2. In neonates submitted to venoarterial ECMO (66) Plasma atrial natriuretic peptide (ANP) presented a nonsignificant decrease during, and significantly increased after ECMO, suggesting a circulating volume contraction with subsequent expansion (66).

3. Amelioration in microcirculatory oxygenation in previously hypoxic and hypoperfused kidney may be associated with ischemia–reperfusion injury (33, 67).

4. About 2% of patients on venoarterial ECMO, presents aortic retrograde dissection that may include renal arteries (68).

5. Systemic thrombotic or air microembolism may cause AKI in patients on venoarterial ECMO.

6. Negative pressure generated by the roller or centrifugal pumps may produce hemolysis and hemoglobinuria (69). Higher pump speed is linked to hemolysis, leukocyte and platelet destruction, and complement activation; To prevent heme pigment-associated AKI, pump revolutions/min must be limited (70, 71).

### ECMO-associated hemostatic derangements

Bleeding and thrombotic complications remain the main causes of morbidity and mortality in ECMO and may coexist in the same patient. Among adult ECMO patients 12.9% develop clots in the oxygenator, and 1.6% develops clots in the bridge, while 15.1% present cannula site bleeding, 14.6% surgical site bleeding, 6% present hemolysis, 3.3% disseminated intravascular coagulation, 2.3% cardiac tamponade and 7.4% pulmonary hemorrhage (43). ECMO circuity thrombosis occur in 13.4% of patients, and thrombosis in 9.5% of patients (72).

ECMO implies blood contact with nonendothelial surfaces that leads to coagulation and fibrinolytic activation and a complement-mediated inflammatory response. Along with blood stasis, physiopathological mechanisms leading to thrombosis in patients on ECMO include (43, 44):

1. Thrombus Deposition in the Circuit and Oxygenator.

2. Pump Thrombosis is rare but clinically relevant;, and may give place to hemolysis.

3. Heparin-induced thrombocytopenia (HIT)

4. Hemolysis; Elevated free hemoglobin in plasma induces *in vitro* Von Willebrand Factor-mediated platelet adhesion, increasing microthrombi generation on fibrinogen, fibrin, extracellular matrix, and collagen at high shear stress. Hemolysis also dysregulates NO, increasing thrombogenesis.

The Mechanisms giving place to bleeding in ECMO patients include (43, 44):

1. Thrombocytopenia.

2. Disseminated Intravascular Coagulation, an acquired disorder characterized by activation of coagulation resulting in fibrin deposition and microthrombi in small and midsized vessels. Associated platelet and coagulation factor consumption may lead to both bleeding or thrombosis.

3. Hyperfibrinolysis. Activation of fibrinolysis may be a primary plasmin-mediated process or a secondary process occurring in the setting of thrombin-mediated activation. Both are commonly seen in extracorporeal life support. Clot deposition on the circuit can lead to excessive fibrinolysis characterized by steep increases in D-dimer and hemorrhage (45).

4. Acquired von Willebrand syndrome (AVWS) is characterized by structural or functional defects in von Willebrand factor typically presenting with mucocutaneous bleeding as well as excessive bleeding after surgery or trauma.

## Extracorporeal liver support

### Extracorporeal liver support multiorgan crosstalk considerations

Among the principal causes of kidney derangements in patients with chronic liver disease, Hepatorenal Syndrome (HS) may present in hepatic failure from any cause. HS patients usually have portal hypertension that triggers arterial vasodilatation in the splanchnic circulation increasing production or activity of vasodilators, being NO of outmost importance. Cardiac output progressively rises with concomitant fall in systemic vascular resistance despite local increases in renal vascular resistances in response to activation of the renin-angiotensin and sympathetic activity (49, 50).

HS is a clinical and laboratory-based construct, being its diagnostic criteria (51):

1. Chronic or acute hepatic disease with hepatic failure and portal hypertension.

2. AKI development with no other cause for AKI.

3. Spontaneous bacterial peritonitis does not exclude the diagnosis of HS.

4. Coexistence of another kidney diagnosis does not exclude hepatorenal syndrome.

5. In conjunction with excluding other apparent causes of kidney disease, the following criteria also apply; Urine red cell excretion <50 cells per high power field, proteinuria <500 mg/day, and lack of improvement in kidney function after volume expansion with intravenous albumin.

HS may be classified as Type 1 (at least a twofold increase in serum creatinine to a level >2.5 mg/dL within less than two weeks) or type 2, less severe, in which the major clinical feature is diuretic-resistant ascites (49, 50).

Diuretics *per se* do not cause hepatorenal syndrome. As cirrhotic patients commonly have poor muscle mass and may present with ascites or anasarca, kidney damage may be worse than suggested by creatinine levels (50).

Important non-HS clinical settings to be taken into account includes prerenal disease, kidney renal manifestations of viral hepatitis, cirrhotic cardiomyopathy, cardiorenal syndrome, corticosteroid deficiency, glomerulonephritis, and acute tubular necrosis (50–52).

### Extracorporeal liver support-associated hemodynamic changes

Increased intrahepatic resistance (IIR), splanchnic vasodilatation, and portosystemic collateral vessels characterize portal hypertension. While vasodilation is present in the mesenteric arterial circulation, intrarenal arteries presents vasoconstriction (53). IIR is mainly because of mechanical factors (regenerative nodules, collagen deposition) and thus irreversible, and about 30% is vasculogenic and reversible (53).

Vasoregulatory pathways responsible for IIR in portal hypertension include Nitric oxide-c-GMP system, aracrine endothelin-1 (ET-1) system, sympathetic alpha adrenergic pathway and angiotensin II (ATII) system. MARS is described to remove this vasoactive substances (73).

In fulminant and acute-on-chronic liver failure, hemodynamic profile includes hyperkinetic circulation including diminished systemic vascular resistance index (SVRI) caused by vasodilation, high cardiac index (CI), and diminished mean arterial pressure (MAP).

Hemodynamic changes in patients with acute-on-chronic liver failure (AOCLF) during the MARS include:

1. Increased heart rate and increased MAP, whereas CI and stroke volume remain without significant changes. Renin levels decrease. (as Renin is not removable by MARS its reduction reflects a decreased release) (74).

2. Decrease in hepatic venous pressure gradient, and decrease in plasma renin activity and norepinephrine (75).

MARS diminishes hyperdynamic circulation (decrease in CO, increased MAP and SVRI), with no significant changes in pulmonary arterial pressure and capillary pulmonary pressures, suggesting no significant change in circulating volume (54).

Compared to Prometheus, MARS is more effective in both decreasing serum bilirubin levels, and improving circulatory variables such as MAP and systemic vascular resistive index, paralleled with diminished plasma renin activity, aldosterone, norepinephrine, vasopressin and nitrate/nitrite levels (54). When compared to albumin-infusion control group, MARS treated patients have been described to significantly decrease both splenic and renal resistive index, accompanied by an increase in portal blood velocity after MARS treatment. Prior to the subsequent session, most parameters had got back to comparable to pretreatment values (76).

### Extracorporeal liver support associated hemostatic derangements

MARS may activate the coagulation system following blood contact with artificial materials, resulting in consumption of coagulation factors and platelets. Bleeding and thrombotic complications are described in g both patients undergoing MARS, and patients with liver failure.

Bleeding complications are specially associated with mortality and can impose a major constraint on liver transplant surgery. Described hemorrhagic complications include severe thrombocytopenia, hypofibrinogenemia, and DIC (55, 56).

1. In a retrospective report, including 83 MARS sessions among 21 patients, 38% of patients presented clinical or laboratory evidence of severe coagulopathy during 12% of the MARS sessions, leading to premature ending of the liver support session in 33% of patients. All coagulation parameters worsened significantly during MARS; median INR augmented significantly, fibrin D-dimer, platelet counts and fibrinogen median decreased (57).

Reported bleeding complications included bleeding from catheter insertion sites, subdural hematoma, severe consumptive coagulopathy with intracranial hematomas and infarctions, and severe esophageal variceal bleed. Reported mortality due to bleeding complications corresponded to 19% of patients treated with MARS, 5% of MARS sessions, and 44% of episodes of severe coagulopathy (57).

2. In a retrospective report on MARS sessions 53.3% of patients presented clinically significant bleeding; with 89% of them presenting muscle or subcutaneous bleeding or hematoma on the catheter insertion site. Coagulation profiles after MARS were concordant with a negative coagulation effect; PT-INR, aPTT, FDP, and D-dimer significantly increased, while platelet and fibrinogen diminished. MARS also reduced coagulation factors, especially factor V (56).

## Cytokines and ECOS

Two of the mayor advantages of ECOS is the capacity to remove toxins and proinflammatory cytokines; this is important given that inseptic shock, dysregulated immune response to infection gives place to cytokine storm, including uncontrolled release of proinflammatory mediators that evoke cellular toxicity (77).

### RRT

Using adsorption membranes, sorbent cartridges or columns during ensue removal of proinflammatory cytokines and endotoxins early in septic shock. Oxiris^®^-AN69 membrane, CytoSorb^®^ and HA380 cytokine hemoadsorption, polymyxin B endotoxin adsorption, and plasmapheresis have been reported to improve clinical outcomes in septic shock, included increased MAP, decreased norepinephrine inotropic score and vasopressor dependency index (36–38).

### ECMO

Similar to the cytokine-mediated inflammatory response to cardiopulmonary bypass, the infiltration of neutrophils is caused by activation of the endothelium and is responsible for the ECMO-related end-organ damage (46–48)., Hemoadsorption during ECMO with good clinical results have been reported, but the need of an extracorporeal circuit for hemoadsorption has considerable limitations (86):

1. Every extra connection in an extracorporeal circuit may lead to accidental air embolism, contamination, or disconnection.

2. Adsorptive drug elimination must be considered.

3. As the limited blood flow allowed through the cartridge needs the integration in a parallel flow setup, it must be taken into account, that Cytosorb^®^ is not approved for high flow circuits with continuous flow rates like the ones used in ECMO.

### Extracorporeal liver support

Liver failure implies compromised hepatic detoxification, protein synthesis, and metabolic derangement with accumulation of a broad spectrum of water-soluble and lipophilic toxins and immune mediators. Oxidized forms of albumin and cytokines, are key factors in the pathogenesis of AOCLF. Pleiotropic properties of albumin make it a plausible therapeutic agent for a physiology-based approach for AOCLF (58, 59).

As bilirubin itself may affect renal functioning *via* bile cast nephropathy, extracorporeal bilirubin removal may represent a kidney protective maneuver (78, 79).

Cytokines and immune mediators implied in hepatic failure can be classified according to its molecular size and biological activity as;

− Smaller proflammatory cytokines like interleukin 6, TNF-α, transforming growth factor-beta 1, and endotoxin components or anaphylatoxins worsens the disease and are negative regulators of hepatocyte proliferation.

− Larger molecules including hepatocyte growth factor, immunoglobulins and components of the complement system improve hepatocyte function and recovery.

During AOCLF MARS has been described to diminish total bilirubin, biliary acids, BUN, ammonia, TNF-alpha, IL-6, and IL-1beta, and its reduction were related to a better clinical prognosis, MARS and Prometheus has been described to have similar serum cytokine clearances (80, 81).

In an *in vitro* two-compartment model comparing MARS albumin dialysis modified with novel charcoal adsorbents to CytoSorb hemoperfusion with added hemodialysis (DIALIVE), both devices had similar performance regarding removal of water-soluble toxins. Ammonia removal was increased using CytoSorb, and CytoSorb lead to a significant reduction of albumin- bound toxins, total bilirubin and subfractions. Bile acid removal was similar while MARS demonstrated no removal of cytokines interleukin-6 and TNF-alpha (61).

## Discussion

Being the kidney a part of a complex multiorgan homeostatic arrangement, any kidney derangement, must be considered according to the whole clinical scenario. Even if multiple clinical characteristics may be shared among groups, every patients represent a unique physiopathological intersectionality to be analyzed.

Multiorgan failure is commonly accompanied by AKI, thus any patient undergoing ECOS should be considered as a patient with high risk of AKI. Being MOF a proinflammatory disease, most physiopathological mechanisms involving kidney damage will include proinflammatory pathways.

Patient interaction with the extracorporeal circuit may affect the kidney function by:

1. Multiorgan crosstalk pathways.

2. Hemodynamic derangements.

3. Coagulation derangements.

4. Cytokine profile and neurohumoral changes.

Expanding the knowledge of ECOS interactions will help to achieve an optimal approach to expected kidney vulnerability or damage scenarios in the course of a successful ECOS (**Table 2**).

**Table 2:** Kidney Vulnerability points during ECOS and its physiopathological mechanisms.

Kidney vulnerability points.	Physiopathological mechanisms.
Hemodynamic derangements.	Diminished intravascular volume. Heart failure. Intravascular/ Interstitial fluid imbalance. Glycocalyx derangements. ECMO-related nonpulsatile blood flow. Ischemia-reperfusion related injury. Renin-angiotensin, sympathetic system and natriuretic peptides disbalance.
Hemostatic (bleeding/thrombotic) derangements.	Intraglomerular bleeding. Thrombocitopenia. Disseminated Intravascular Coagulation. Hyperfibrinolysis. Acquired von Willebrand syndrome. Renal arteries dissection.
Multiorgan crosstalk.	Preexisting CKD systemic consequences. Cardiorenal syndrome. Hepatorenal Syndrome. Lung-Kidney interactions. Systemic proinflammatory milieu. Systemic repercussions of endotoxin and toxins accumulation.

## Author contributions

The author confirms being the sole contributor of this work and has approved it for publication.

## Conflict of interest

The author declares that the research was conducted in the absence of any commercial or financial relationships that could be construed as a potential conflict of interest.

## Publisher’s note

All claims expressed in this article are solely those of the authors and do not necessarily represent those of their affiliated organizations, or those of the publisher, the editors and the reviewers. Any product that may be evaluated in this article, or claim that may be made by its manufacturer, is not guaranteed or endorsed by the publisher.

## Glossary

**Table:**

ADH	Antidiuretic hormone
ADH	Antidiuretic hormone
AKI	Acute kidney injury
ANP	Atrial natriuretic peptide
ANP	Plasma atrial natriuretic peptide
AOCLF	acute-on-chronic liver failure AOCLF, acute-on-chronic liver failure
ARDS	acute respiratory distress syndrome
aRRT	Acute renal replacement therapy
AVWS	Acquired von Willebrand syndrome
CI	cardiac index
CI	cardiac index
CKD	Chronic kidney disease
CVP	Central venous pressure
DIC	disseminated intravascular coagulation
ECMO	Extracorporeal membrane oxygenation
ECOS	Extracorporeal organ support
ELSO	Extracorporeal Life Support Organisation
ER	Endoplasmic reticulum
ESKD	End-stage kidney disease
FHF	Fulminant hepatic failure
GFR	GLomerular filtration rate
HIRRT	Hemodynamic instability during renal replacement therapy continuous renal replacement therapy
HIT	Heparin-induced thrombocytopenia
HS	Hepatorenal Syndrome
IHVR	Intrahepatic vascular resistance
IIR	Increased intrahepatic resistance
MAP	mean arterial pressure
MAP	mean arterial pressure
MARS	molecular adsorbents recirculating system
MARS	molecular adsorbents recirculating system
MOF	Multiple organ failure
NO	nitric oxide
ROS	reactive oxygen species
PAMP	Pathogen-associated molecular patterns
PRA	Plasma renin activity
SVRI	Systemic vascular resistance index

## References

[1] Stange J. Extracorporeal liver support. Organogenesis. (2011) 7(1):64–73. doi: 10.4161/org.7.1.14069 21343699 PMC3082035

[2] De Rosa S Samoni S Villa G Ronco C. Management of chronic kidney disease patients in the intensive care unit: Mixing acute and chronic illness. Blood Purif (2017) 43(1–3):151–62. doi: 10.1159/000452650 28114127

[3] Chawla LS Eggers PW Star RA Kimmel PL. Acute kidney injury and chronic kidney disease as interconnected syndromes. N Engl J Med (2014) 371(1):58–66. doi: 10.1056/NEJMra1214243 24988558 PMC9720902

[4] Maekawa H Inagi R. Pathophysiological role of organelle stress/crosstalk in AKI to CKD transition. Semin Nephrol (2019) 39(6):581–8. doi: 10.1016/j.semnephrol.2019.10.007 31836040

[5] Cybulsky AV. The intersecting roles of endoplasmic reticulum stress, ubiquitin proteasome system, and autophagy in the pathogenesis of proteinuric kidney disease. Kidney Int (2013) 84(1):25–33. doi: 10.1038/ki.2012.390 23254900

[6] Li Y Zhang Y Wang J Chen W Cai Y Yang M. Pulmonary hypertension in end stage renal disease patients on dialysis and predialysis patients. Clin Invest Med (2020) 43(3)::E4448. doi: 10.25011/cim.v43i3.34631 32971584

[7] Ewert R Opitz C Wensel R Dandel M Mutze S Reinke P. Abnormalities of pulmonary diffusion capacity in long term survivors after kidney transplantation. Chest. (2002) 122(2):639–44. doi: 10.1378/chest.122.2.639 12171844

[8] Husain Syed F McCullough PA Birk HW Renker M Brocca A Seeger W. Cardio pulmonary renal interactions: A multidisciplinary approach. J Am Coll Cardiol (2015) 65(22):2433–48. doi: 10.1016/j.jacc.2015.04.024 26046738

[9] Gross ML Ritz E. Hypertrophy and fibrosis in the cardiomyopathy of uremia beyond coronary heart disease: Cardiac hypertrophy and fibrosis in renal insufficiency. Semin Dial. (2008) 21(4):308–18. doi: 10.1111/j.1525139X.2008.00454.x 18627569

[10] Ronco C Cicoira M McCullough PA. Cardiorenal syndrome type 1: pathophysiological crosstalk leading to combined heart and kidney dysfunction in the setting of acutely decompensated heart failure. J Am Coll Cardiol (2012) 60(12):1031–42. doi: 10.1016/j.jacc.2012.01.077 22840531

[11] Ito T. PAMPs and DAMPs as triggers for DIC. J Intensive Care (2014) 2(1):67. doi: 10.1186/s4056001400650 PMC4336279 25705424

[12] Villa G Husain Syed F Saitta T Degl’Innocenti D Barbani F Resta M. Hemodynamic instability during acute kidney injury and acute renal replacement therapy: Pathophysiology and clinical implications. Blood Purif (2021) 50(6):729–39. doi: 10.1159/000513942 33756481

[13] Douvris A Zeid K Hiremath S Bagshaw SM Wald R Beaubien Souligny W. Mechanisms for hemodynamic instability related to renal replacement therapy: a narrative review. Intensive Care Med (2019) 45(10):1333–46. doi: 10.1007/s0013401905707w PMC6773820 31407042

[14] Uchino S Bellomo R Morimatsu H Morgera S Schetz M Tan I. Continuous renal replacement therapy: a worldwide practice survey. the beginning and ending supportive therapy for the kidney (B.E.S.T. kidney) investigators: The beginning and ending supportive therapy for the kidney (B.E.S.T. kidney) investigators. Intensive Care Med (2007) 33(9):1563–70. doi: 10.1007/s0013400707544 17594074

[15] Akhoundi A Singh B Vela M Chaudhary S Monaghan M Wilson GA. Incidence of adverse events during continuous renal replacement therapy. Blood Purif (2015) 39(4):333–9. doi: 10.1159/000380903 26022612

[16] National Kidney Foundation. KDOQI clinical practice guideline for hemodialysis adequacy: 2015 update. Am J Kidney Dis (2015) 66(5):884–930. doi: 10.1053/j.ajkd.2015.07.015 26498416

[17] Pun PH Horton JR Middleton JP. Dialysate calcium concentration and the risk of sudden cardiac arrest in hemodialysis patients. Clin J Am Soc Nephrol (2013) 8(5):797–803. doi: 10.2215/CJN.10000912 23371957 PMC3641623

[18] Yagi N Leblanc M Sakai K Wright EJ Paganini EP. Cooling effect of continuous renal replacement therapy in critically ill patients. Am J Kidney Dis (1998) 32(6):1023–30. doi: 10.1016/s02726386(98)700782 9856519

[19] Dietrichs ES Sager G Tveita T. Altered pharmacological effects of adrenergic agonists during hypothermia. Scand J Trauma Resusc Emerg Med (2016) 24(1):143. doi: 10.1186/s1304901603398 27919274 PMC5139099

[20] Piotti A Novelli D Meessen JMTA Ferlicca D Coppolecchia S Marino A. Endothelial damage in septic shock patients as evidenced by circulating syndecan 1, sphingosine 1 phosphate and soluble VE cadherin: a substudy of ALBIOS. Crit Care (2021) 25(1):113. doi: 10.1186/s13054021035451 33741039 PMC7980645

[21] Hesselvik JF Malm J Dahlbäck B Blombäck M. Protein s and C4b binding protein in severe infection and septic shock. Thromb Haemost. (1991) 65(2):126–9. 1828915

[22] Cardigan RA McGloin H Mackie IJ Machin SJ Singer M. Activation of the tissue factor pathway occurs during continuous venovenous hemofiltration. Kidney Int (1999) 55(4):1568–74. doi: 10.1046/j.15231755.1999.00397.x 10201024

[23] Wand S Schneider S Meybohm P Zacharowski K Weber CF. Assessment of hemostatic changes after initiation of continuous venovenous hemodialysis. Clin Lab (2015) 61(3–4):379–87. doi: 10.7754/clin.lab.2014.140931 25975006

[24] Davies H Leslie G. Maintaining the CRRT circuit: non anticoagulant alternatives. Aust Crit Care (2006) 19(4):133–8. doi: 10.1016/s10367314(06)800263 17165492

[25] Zarka F Tayler Gomez A Ducruet T Duca A Albert M Bernier Jean A. Risk of incident bleeding after acute kidney injury: A retrospective cohort study. J Crit Care (2020) 59:23–31. doi: 10.1016/j.jcrc.2020.05.003 32485439

[26] Zarbock A Küllmar M Kindgen Milles D Wempe C Gerss J Brandenburger T. Effect of regional citrate anticoagulation vs systemic heparin anticoagulation during continuous kidney replacement therapy on dialysis filter life span and mortality among critically ill patients with acute kidney injury: A randomized clinical trial: A randomized clinical trial. JAMA. (2020) 324(16):1629–39. doi: 10.1001/jama.2020.18618 PMC7585036 33095849

[27] Brodsky S Eikelboom J Hebert LA. Anticoagulant related nephropathy. J Am Soc Nephrol (2018) 29(12):2787–93. doi: 10.1681/ASN.2018070741 PMC6287865 30420420

[28] L’Imperio V Guarnieri A Pieruzzi F Sinico RA Pagni F. Anticoagulant related nephropathy: a pathological note. J Thromb Thrombolysis (2018) 46(2):260–3. doi: 10.1007/s11239-018-1669-3 29679257

[29] Zeni L Manenti C Fisogni S Terlizzi V Verzeletti F Gaggiotti M. Acute kidney injury due to anticoagulant related nephropathy : A suggestion for therapy. Case Rep Nephrol (2020) 2020:8952670. doi: 10.1155/2020/8952670 32566333 PMC7298278

[30] Cybulsky AV. Endoplasmic reticulum stress, the unfolded protein response and autophagy in kidney diseases. Nat Rev Nephrol (2017) 13(11):681–96. doi: 10.1038/nrneph.2017.129 28970584

[31] Braun D Dietze S Pahlitzsch TMJ Wennysia IC Persson PB Ludwig M. Short term hypoxia and vasa recta function in kidney slices. Clin Hemorheol Microcirc (2017) 67(3–4):475–84. doi: 10.3233/CH179230 28922144

[32] Joannidis M Forni LG Klein SJ Honore PM Kashani K Ostermann M. Lung kidney interactions in critically ill patients: consensus report of the acute disease quality initiative (ADQI) 21 workgroup. Intensive Care Med (2020) 46(4):654–72. doi: 10.1007/s00134019058697 PMC7103017 31820034

[33] Ostermann M Lumlertgul N. Acute kidney injury in ECMO patients. Crit Care (2021) 25(1):313. doi: 10.1186/s13054021036765 34461966 PMC8405346

[34] Kilburn DJ Shekar K Fraser JF. The complex relationship of extracorporeal membrane oxygenation and acute kidney injury: Causation or association? BioMed Res Int (2016) 2016:1094296. doi: 10.1155/2016/1094296 27006941 PMC4783537

[35] Ma J Yuan HX Chen YT Ning DS Liu XJ Peng YM. Circulating endothelial microparticles: a promising biomarker of acute kidney injury after cardiac surgery with cardiopulmonary bypass. Ann Transl Med (2021) 9(9):786. doi: 10.21037/atm207828 34268399 PMC8246187

[36] Lumlertgul N Srisawat N. The haemodynamic effects of oXiris haemofilter in septic shock patients requiring renal support: A single centre experience. Int J Artif Organs. (2021) 44(1):17–24. doi: 10.1177/0391398820917150 32393096

[37] Yang Q Li Y Tuohuti P Qin Z Zhang Z Zhao W. Advances in the development of biomaterials for endotoxin adsorption in sepsis. Front Bioeng Biotechnol (2021) 9:699418. doi: 10.3389/fbioe.2021.699418 34395405 PMC8361450

[38] Shum HP KC C MC K Yan WW. Application of endotoxin and cytokine adsorption haemofilter in septic acute kidney injury due to gram negative bacterial infection. Hong Kong Med J (2013) 19(6):491–7. doi: 10.12809/hkmj133910 23650198

[39] Jung S Bosch A Kolwelter J Striepe K Kannenkeril D Schuster T. Renal and intraglomerular haemodynamics in chronic heart failure with preserved and reduced ejection fraction. ESC Heart Fail (2021) 8(2):1562–70. doi: 10.1002/ehf2.13257 PMC8006684 33559346

[40] Mårtensson J Bellomo R. Sepsis induced acute kidney injury. Crit Care Clin (2015) 31(4):649–60. doi: 10.1016/j.ccc.2015.06.003 26410135

[41] O’Neil MP Fleming JC Badhwar A Guo LR. Pulsatile versus nonpulsatile flow during cardiopulmonary bypass: microcirculatory and systemic effects. Ann Thorac Surg (2012) 94(6):2046–53. doi: 10.1016/j.athoracsur.2012.05.065 22835552

[42] Orime Y Shiono M Hata H Yagi S Tsukamoto S Okumura H. Cytokine and endothelial damage in pulsatile and nonpulsatile cardiopulmonary bypass. Artif Organs. (1999) 23(6):508–12. doi: 10.1046/j.15251594.1999.06392.x 10392275

[43] Zeni L Manenti C Fisogni S Terlizzi V Verzeletti F Gaggiotti M. Acute kidney injury due to anticoagulant related nephropathy : A suggestion for therapy. Case Rep Nephrol (2020) 2020:8952670. doi: 10.1155/2020/8952670 32566333 PMC7298278

[44] Thomas J Kostousov V Teruya J. Bleeding and thrombotic complications in the use of extracorporeal membrane oxygenation. Semin Thromb Hemost (2018) 44(1):20–9. doi: 10.1055/s00371606179 28898902

[45] Hunt BJ Parratt RN Segal HC Sheikh S Kallis P Yacoub M. Activation of coagulation and fibrinolysis during cardiothoracic operations. Ann Thorac Surg (1998) 65(3):712 8. doi: 10.1016/s00034975(97)013453 9527200

[46] McILwain RB Timpa JG Kurundkar AR Holt DW Kelly DR Hartman YE. Plasma concentrations of inflammatory cytokines rise rapidly during ECMO related SIRS due to the release of preformed stores in the intestine. Lab Invest (2010) 90(1):128–39. doi: 10.1038/labinvest.2009.119 PMC2799549 19901912

[47] Millar JE Fanning JP McDonald CI McAuley DF Fraser JF. The inflammatory response to extracorporeal membrane oxygenation (ECMO): a review of the pathophysiology. Crit Care (2016) 20(1):387. doi: 10.1186/s1305401615704 27890016 PMC5125043

[48] Datzmann T Träger K. Extracorporeal membrane oxygenation and cytokine adsorption. J Thorac Dis (2018) 10(Suppl 5):S653–60. doi: 10.21037/jtd.2017.10 PMC5911550 29732183

[49] Ginès A Escorsell A Ginès P Saló J Jiménez W Inglada L. Incidence, predictive factors, and prognosis of the hepatorenal syndrome in cirrhosis with ascites. Gastroenterology. (1993) 105(1):229–36. doi: 10.1016/00165085(93)900317 8514039

[50] Muciño Bermejo J Carrillo Esper R Uribe M Méndez Sánchez N. Acute kidney injury in critically ill cirrhotic patients: a review. Ann Hepatol (2012) 11(3):301–10. doi: 10.1016/s16652681(19)30924x 22481447

[51] Biggins SW Angeli P Garcia Tsao G Ginès P Ling SC Nadim MK. Diagnosis, evaluation, and management of ascites, spontaneous bacterial peritonitis and hepatorenal syndrome: 2021 practice guidance by the American association for the study of liver diseases. Hepatology. (2021) 74(2):1014–48. doi: 10.1002/hep.31884 33942342

[52] Muciño Bermejo MJ. Mechanisms of kidney dysfunction in the cirrhotic patient: Non hepatorenal acute on chronic kidney damage considerations. Ann Hepatol (2020) 19(2):145–52. doi: 10.1016/j.aohep.2019.06.022 31594758

[53] Schrier RW Arroyo V Bernardi M Epstein M Henriksen JH Rodés J. Peripheral arterial vasodilation hypothesis: a proposal for the initiation of renal sodium and water retention in cirrhosis. Hepatology. (1988) 8(5):1151–7. doi: 10.1002/hep.1840080532 2971015

[54] Laleman W Wilmer A Evenepoel P Elst IV Zeegers M Zaman Z. Effect of the molecular adsorbent recirculating system and Prometheus devices on systemic haemodynamics and vasoactive agents in patients with acute on chronic alcoholic liver failure. Crit Care (2006) 10(4):R108. doi: 10.1186/cc4985 16859530 PMC1751025

[55] Stravitz RT Ellerbe C Durkalski V Schilsky M Fontana RJ Peterseim C. Bleeding complications in acute liver failure. Hepatology. (2018) 67(5):1931–42. doi: 10.1002/hep.29694 PMC5906191 29194678

[56] Yoo SW Ki MJ Kim D Kim SK Park S Han HJ. Bleeding complications associated with the molecular adsorbent recirculating system: a retrospective study. Acute Crit Care (2021) 36(4):322–31. doi: 10.4266/acc.2021.00276 PMC8907459 35263827

[57] Bachli EB Schuepbach RA Maggiorini M Stocker R Müllhaupt B Renner EL. Artificial liver support with the molecular adsorbent recirculating system: activation of coagulation and bleeding complications. Liver Int (2007) 27(4):475–84. doi: 10.1111/j.14783231.2006.01398.x 17403187

[58] Clària J Stauber RE Coenraad MJ Moreau R Jalan R Pavesi M. Systemic inflammation in decompensated cirrhosis: Characterization and role in acute on chronic liver failure. Hepatology. (2016) 64(4):1249–64. doi: 10.1002/hep.28740 27483394

[59] Bernardi M Angeli P Claria J Moreau R Gines P Jalan R. Albumin in decompensated cirrhosis: new concepts and perspectives. Gut. (2020) 69(6):1127–38. doi: 10.1136/gutjnl2019318843 PMC7282556 32102926

[60] Rozga J Umehara Y Trofimenko A Sadahiro T Demetriou AA. A novel plasma filtration therapy for hepatic failure: preclinical studies. Ther Apher Dial. (2006) 10(2):138–44. doi: 10.1111/j.1744-9987.2006.00355.x 16684215

[61] Dominik A Stange J. Similarities, differences, and potential synergies in the mechanism of action of albumin dialysis using the MARS albumin dialysis device and the CytoSorb hemoperfusion device in the treatment of liver failure. Blood Purif (2021) 50(1):119–28. doi: 10.1159/000508810 32615564

[62] Gu M Mei XL Zhao YN. A review on extracorporeal membrane oxygenation and kidney injury. J Biochem Mol Toxicol (2021) 35(3):e22679. doi: 10.1002/jbt.22679 33325616

[63] Mao H Katz N Kim JC Day S Ronco C. Implantable left ventricular assist devices and the kidney. Blood Purif (2014) 37(1):57–66. doi: 10.1159/000357970 24525434

[64] Gori M Senni M Gupta DK Charytan DM Kraigher Krainer E Pieske B. Association between renal function and cardiovascular structure and function in heart failure with preserved ejection fraction. Eur Heart J (2014) 35(48):3442–51. doi: 10.1093/eurheartj/ehu254 PMC4810804 24980489

[65] Villa G Katz N Ronco C. Extracorporeal membrane oxygenation and the kidney. Cardiorenal Med (2015) 6(1):50–60. doi: 10.1159/000439444 27194996 PMC4698639

[66] Semmekrot BA Pesman GJ Span PN Sweep CGJ van Heijst AFJ Monnens LAH. Serial plasma concentrations of atrial natriuretic peptide, plasma renin activity, aldosterone, and antidiuretic hormone in neonates on extracorporeal membrane oxygenation. ASAIO J (2002) 48(1):26–33. doi: 10.1097/0000248020020100000007 11820219

[67] Karagiannidis C Hesselmann F Fan E. Physiological and technical considerations of extracorporeal CO2 removal. Crit Care (2019) 23(1):75. doi: 10.1186/s130540192367z 30849995 PMC6408850

[68] Cheng R Hachamovitch R Kittleson M Patel J Arabia F Moriguchi J. Complications of extracorporeal membrane oxygenation for treatment of cardiogenic shock and cardiac arrest: a meta analysis of 1,866 adult patients. Ann Thorac Surg (2014) 97(2):610–6. doi: 10.1016/j.athoracsur.2013.09.008 24210621

[69] MacLaren G Combes A Bartlett RH. Contemporary extracorporeal membrane oxygenation for adult respiratory failure: life support in the new era. Intensive Care Med (2012) 38(2):210–20. doi: 10.1007/s0013401124392 22147116

[70] Lou S MacLaren G Best D Delzoppo C Butt W. Hemolysis in pediatric patients receiving centrifugal pump extracorporeal membrane oxygenation: prevalence, risk factors, and outcomes. Crit Care Med (2014) 42(5):1213–20. doi: 10.1097/CCM.0000000000000128 24351369

[71] Salis S Mazzanti VV Merli G Salvi L Tedesco CC Veglia F. Cardiopulmonary bypass duration is an independent predictor of morbidity and mortality after cardiac surgery. J Cardiothorac Vasc Anesth (2008) 22(6):814 22. doi: 10.1053/j.jvca.2008.08.004 18948034

[72] Olson SR Murphree CR Zonies D Meyer AD Mccarty OJT Deloughery TG. Thrombosis and bleeding in extracorporeal membrane oxygenation (ECMO) without anticoagulation: A systematic review. ASAIO J (2021) 67(3):290–6. doi: 10.1097/MAT.0000000000001230 PMC8623470 33627603

[73] Bhathal PS Grossman HJ. Reduction of the increased portal vascular resistance of the isolated perfused cirrhotic rat liver by vasodilators. J Hepatol (1985) 1(4):325–37. doi: 10.1016/s01688278(85)807704 4056346

[74] Schmidt LE Sørensen VR Svendsen LB Hansen BA Larsen FS. Hemodynamic changes during a single treatment with the molecular adsorbents recirculating system in patients with acute on chronic liver failure. Liver Transpl (2001) 7(12):1034–9. doi: 10.1053/jlts.2001.29108 11753905

[75] Catalina MV Barrio J Anaya F Salcedo M Rincón D Clemente G. Hepatic and systemic haemodynamic changes after MARS in patients with acute on chronic liver failure. Liver Int (2003) 23 Suppl 3:39–43. doi: 10.1034/j.14783231.23.s.3.10.x 12950960

[76] Donati G Piscaglia F Colì L Silvagni E Righini R Donati G. Acute systemic, splanchnic and renal haemodynamic changes induced by molecular adsorbent recirculating system (MARS) treatment in patients with end stage cirrhosis: Mars and haemodynamic changes in cirrhosis. Aliment Pharmacol Ther (2007) 26(5):717–26. doi: 10.1111/j.1365-2036.2007.03420.x 17697205

[77] Hellman T Uusalo P Järvisalo MJ. Renal replacement techniques in septic shock. Int J Mol Sci (2021) 22(19):10238. doi: 10.3390/ijms221910238 34638575 PMC8508758

[78] Krones E Pollheimer MJ Rosenkranz AR Fickert P. Cholemic nephropathy historical notes and novel perspectives. Biochim Biophys Acta Mol Basis Dis (2018) 1864(4 Pt B):1356–66. doi: 10.1016/j.bbadis.2017.08.028 28851656

[79] Sens F Bacchetta J Rabeyrin M Juillard L. Efficacy of extracorporeal albumin dialysis for acute kidney injury due to cholestatic jaundice nephrotoxicity. BMJ Case Rep (2016) 2016. doi: 10.1136/bcr-2015-213257 PMC4957002 27389722

[80] Stadlbauer V Krisper P Aigner R Haditsch B Jung A Lackner C. Effect of extracorporeal liver support by MARS and Prometheus on serum cytokines in acute on chronic liver failure. Crit Care (2006) 10(6):R169. doi: 10.1186/cc5119 17156425 PMC1794485

[81] Di Campli C MA Z Gaspari R Novi M Candelli M Santoliquido A. The decrease in cytokine concentration during albumin dialysis correlates with the prognosis of patients with acute on chronic liver failure. Transplant Proc (2005) 37(6):2551–3. doi: 10.1016/j.transproceed.2005.06.040 16182740

